# Recent advances in managing/understanding meningioma

**DOI:** 10.12688/f1000research.13674.1

**Published:** 2018-04-24

**Authors:** Nawal Shaikh, Karan Dixit, Jeffrey Raizer

**Affiliations:** 1 Northwestern University Feinberg School of Medicine, Chicago, USA

**Keywords:** meningiomas, management, imaging, genetics

## Abstract

Meningiomas are the most common adult primary intracranial tumor. Despite their higher incidence, there have not—until recently—been as many advances in understanding and managing meningiomas. Thus far, two broad classes of meningiomas have emerged on the basis of their mutational profile: those driven by neurofibromatosis 2 (NF2) inactivation and those with non-NF2 driver gene alterations, such as mammalian target of rapamycin and Hedgehog, Wingless/b-catenin, Notch, transforming growth factor-b receptor, mitogen-activated protein kinase, and phospholipase C pathway alterations. In addition to improvements in molecular diagnostics, advances in imaging are being studied to better predict tumor behavior, stratify risk, and potentially monitor for disease response. Management consists primarily of surgery and radiation therapy and there has been limited success from medical therapies, although novel targeted agents are now in clinical trials. Advances in imaging and understanding of the genetic makeup of meningiomas demonstrate the huge potential in revolutionizing the classification, diagnosis, management, and prognosis of meningiomas..

## Introduction

Meningiomas are dural-based tumors that arise from arachnoid cap or meningothelial cells. They are the most common adult primary intracranial tumor. Despite their higher incidence, there have not—until recently—been as many advances in understanding and managing meningiomas. Meningiomas are usually slow-growing tumors; however, there are more aggressive, but less common, subtypes ^1^. Some benign meningiomas (BMs) follow a more aggressive course with multiple recurrences, whereas some atypical meningiomas (AMs) and malignant meningiomas (MMs) can have a rather benign course with long progression-free survival (PFS) and overall survival (OS). Counterintuitively, it has been realized for some time that the tumor characteristics associated with high-grade histopathology and those associated with recurrence/progression do not always correlate ^2^. Hence, much work is being performed in the clinic, as well as in the fields of advanced imaging and genomics, to discover other features or phenomena that contribute to tumor growth and recurrence.

## Epidemiology and grading

Meningiomas account for approximately 37% of all primary central nervous system tumors in the US. The incidence increases with age and there is a notable increase after the age of 65. They are nearly twice as common in females than in males and are estimated to be three times more common in females within the age range of 35 to 54 years.

The World Health Organization (WHO) classification for meningiomas is based solely on histopathological characterizations of mitotic rate, cellular features of atypia, and local invasion. About 80% are WHO grade I (also referred to as BM), 17% are WHO grade II (AM), and 2% are WHO grade III (anaplastic meningioma/MM) ^3^. The WHO classification has prognostic value but has limitations because of a lack of reliable molecular markers for aggressive and recurrence-prone tumors ^4, 5^.

## Molecular genetics

The transformed classification and subsequent management and prognostication of several brain tumor types, such as glioblastoma, ependymoma, and medulloblastoma, exemplify the paradigm shift toward molecular taxonomy, which is now being applied to meningiomas with high-throughput genomic and epigenomic analyses. Many groups have used next-generation sequencing as a tool to render a genetic element to better define and classify meningiomas and consequently find novel therapeutic targets to complement surgery and radiation.

The concept of genetic contribution to the causation of meningiomas has been derived from associated familial syndromes. The first and most widely described of these syndromes is neurofibromatosis 2 (NF2), in which 50 to 75% of patients develop one or more meningiomas ^6^. The *NF2* gene is a tumor suppressor present on chromosome 22 and encodes for a protein called merlin (also known as schwannomin), which plays a key role in regulating meningioma cell proliferation and tumor formation in mouse models and in regulating multiple downstream pathways ^7^. Merlin links the actin cytoskeleton to plasma membrane proteins and is an important inhibitor of contact-dependent proliferation. Loss of merlin, which occurs in NF2 mutations, leads to increased YAP expression and increases cell proliferation from loss of contact-dependent inhibition ^8^. Mutations in *NF2* gene are found in 50–60% of patients with BM and in up to 75% of patients with AM and MM. *NF2* mutated meningiomas are more prone to tumor progression as the gene has been found more frequently mutated and associated with multiple allelic losses in the most aggressive meningiomas. Interestingly, many genes, including *NF2, BAM22, INI1, TIMP-1*, and *DAL-1*, are mutated with chromosome 22 loss ^9– 12^.

Thus far, two broad classes of meningiomas have emerged on the basis of their mutational profile: those driven by *NF2* inactivation and those with non-NF2 driver gene alterations, such as mammalian target of rapamycin (mTOR) and Hedgehog, Wingless (WNT)/b-catenin, Notch, transforming growth factor-b receptor (TGF-bR), mitogen-activated protein kinase (MAPK), and phospholipase C pathway alterations.

In about 20% of meningiomas, no genetic alterations are detected. In these meningiomas as well as those with defined mutations, epigenomic alterations may play an important role in tumor development and progression. Of the epigenomic alterations, methylation is the most thoroughly studied. For instance, RIZ1 expression negatively correlates with tumor grade; grade I, II, and III meningiomas express RIZ1 in 87.5%, 38.9%, and 23.8% of cases, respectively. Comethylation of several homeobox (HOX) genes has been implicated in the tumorigenesis of high-grade meningiomas. Aberrant hypermethylation of *WNK2* is associated with 83% and 71% of grade II and III meningiomas. Notably, a scoring system based on quantified methylation values of five genes ( *HOXA6, HOXA9, PENK, UPK3A*, and *IGF2bP1*) was reported to provide 80 to 90% sensitivity and specificity in predicting recurrence of meningiomas, independent of tumor grade ^6^. Olar *et al*. demonstrated the robust ability to stratify meningiomas in 140 samples by using global DNA methylation signatures to accurately identify patients with tumors more likely to recur ^13^. This method could be used in conjunction with clinical and histologic grading scales to risk-stratify patients who require more aggressive upfront therapy with radiation ^13^. This idea was further reinforced by the Heidelberg, Germany group led by Sahm *et al*., who also investigated genome-wide DNA methylation patterns of 497 meningiomas in a retrospective analysis and concluded that, compared with the current WHO classification, the DNA methylation-based meningioma classification is able to segregate meningiomas in more homogenous groups in terms of predicting tumor recurrence and prognosis ^4^.

Genomic instability is one of the important differentiators between grade I and grade II or III meningiomas ^6^. The Dana-Farber group found from their *in vitro* meningioma cell lines that loss of chromosome 22 is the most common arm-level alteration across all meningiomas (40–60% in grade I and 75% in grade II or III), along with recurrent loss of chromosomes 1p, 6q, 10q, 14q, and 18q and gain of 1q, 9q, 12q, 15q, 17q, and 20q in high-grade tumors. Among these, chromosome 1p and 14q loss are the most frequent cytogenetic abnormalities observed in meningiomas after chromosome 22, affecting half of all grade II and nearly all grade III meningiomas ^14^. A higher level of genomic disruption was identified in the two high-grade meningioma cell lines, consistent with the original meningioma being of a high-grade nature ^15^.

Targeting of telomerases is an exciting area of research in regulating cancer senescence. Telomerase activation has been demonstrated in 10% of grade I, 50% of grade II, and 95% of grade III meningiomas ^6^. Hence, targeted telomerase inhibitors may have potential in treating meningiomas ^16^.

Alternative methods of meningioma regulation have been seen in the impact of microRNA molecules (miRs), which are small nucleotide sequences involved in the suppression of mRNA translation. Importantly, inducing or suppressing these molecules has been considered among the approaches in the treatment of tumors. Several miRs involved in regulating meningioma proliferation include miR-200a ^17^ and miR-224 ^18^. One study showed dysregulation of 13 miRs in BMs and 52 miRs in anaplastic meningioma ^19^.

To further expand the young genetic landscape of meningioma, Tang *et al*. performed whole genome sequencing across seven tumor-normal pairs to identify somatic genetic alterations in meningioma ^20^. The majority of copy number variants and single-nucleotide variants were chromatin regulators, including multiple histone members, histone-modifying enzymes, and several epigenetic regulators. Recurrent chromosomal arrangements on chromosome 22q, 6p, and 1q were detected ^20^.

## Imaging

Imaging characteristics, advanced imaging technology, and radiomics are playing an increasingly important role in tumor diagnosis, prognosis, and treatment response. Diffusion-weighted magnetic resonance imaging (MRI), diffusor tensor imaging, and positron emission tomography (PET) imaging have been studied for preoperative prediction of biological behavior of meningiomas; however, their clinical utility is not yet established. Peritumoral edema around meningiomas has been associated with higher proliferation index and irregular tumor margins which may be a marker for more aggressive phenotype ^21^. Increased vascular endothelial growth factor (VEGF) secretion and associated angiogenesis may also be associated with peritumoral edema ^22^. Comprehensive risk stratification models deploying imaging features such as preoperative apparent diffusion coefficient MRI sequences, along with Simpson grade of classification, have shown superiority in envisaging which patients will experience progression/recurrence over standard histopathological grading and histopathological in combination with Simpson grading ^23^. Recently, a retrospective analysis of a small cohort of patients showed that preoperative fractal analysis of MRIs, a software method which better describes complexity of an image, may play a role in identifying non-BMs ^1, 24– 26^.

Since the 1990s, octreotide scintigraphy has been demonstrated as an effective method to image meningiomas ^27^. More contemporary imaging techniques such as PET imaging have added a new dimension in the diagnosis and grading of meningiomas. Gadolinium DOTA-octreotate ( ^68^Ga-DOTATATE) PET has been shown to be a reliable predictor of tumor growth in BM and AM. Moreover, tumors with fast growth rate and transosseous expansion have the highest binding of the radionuclide, which indicates the potential for DOTATATE-based therapy ^28^.

## Management

Surgery and radiation therapy (RT) have been the cornerstone of treatment for meningiomas of all grades. Chemotherapy thus far has shown limited benefit on the basis of several retrospective studies; however, with increasing understanding of molecular pathways, there may be a greater role for targeted drugs. An important aspect in the management of meningioma is predicting the risk of recurrence. With the publishing of the Simpson grading scale in 1957, the extent of resection has been a central component of meningioma management and predictive of recurrence ^29^. Tumor location has also been considered a predictor of recurrence ^30^. Skull-based meningiomas, which are commonly benign, are an example of location negatively impacting recurrence-free survival/PFS because the deep location and relation to surrounding critical neurovascular structures often limit the extent of resection and residual tumor increases the risk of recurrence ^31, 32^. Male gender, lack of calcification, reduced expression of chromosome 1p, VEGF expression, and MIB-L1 (monoclonal antibody tumor proliferation marker) are other factors associated with meningioma recurrence ^29, 30^.

Small, asymptomatic presumed meningiomas can be followed conservatively by observation and periodic imaging, and surgical intervention can be pursued if patients become symptomatic or there is significant growth ^33^. Surgery with the goal of gross total resection (GTR) is the treatment of choice for symptomatic meningiomas. The estimated 10-year PFS rates are about 60–80% for gross total resected WHO grade I meningiomas and 50% for those with subtotal resection (STR) ^34^. Gross totally resected BMs can be followed with serial imaging. The risk of recurrence for subtotally resected BMs is about 40–50% at five years; thus, adjuvant radiation can be considered for tumors in critical areas such as the skull base and near venous sinuses ^35^. Stereotactic radiosurgery (SRS) has also been used as first-line therapy, usually in patients whose meningioma presents significant surgical challenges ^36– 38^. These challenges are related to tumor location, patient age, comorbidities, recurrence after incomplete resection, and risks of neurologic morbidity if resection is pursued ^39^. Local control rates are best for tumors less than 10 cm ^3^ in volume. The five-year outcomes for BM treated with SRS versus surgical resection are nearly similar. Longer-term (10-year) follow-up of BMs after SRS has revealed a broad range of tumor control rates between 69 and 92% with more recent data ^36, 39– 41^. Though non-invasive when compared with surgery, SRS is associated with potential toxicities, including cranial neuropathies from RT-induced injury. To avoid these complications in vital structures such as the optic nerve, the standard minimum distance between the meningioma and anterior optic apparatus is 5 mm; however, with modern radiosurgical technology and hypofractionated SRS regimens, the distance has been decreased to nearly zero ^39^. Adaptive hybrid approaches of near total resection followed by SRS for meningiomas located in critical areas near important vascular and neural structures are increasingly being used ^39^.

The initial management for AM (WHO grade II) is surgery with a goal of maximal safe resection if possible. Several retrospective analyses have demonstrated the importance of GTR for AMs: five-year PFS rates were 60–90% after GTR and 30–70% after STR ^34, 35, 42– 45^. The 10-year PFS has been estimated at 87% for GTR but only 17% for STR ^46^. The benefit of adjuvant external beam radiation after GTR for AM is debated; prior retrospective studies show mixed results of early adjuvant RT following GTR and thus the current recommendation is active surveillance ^43, 47, 48^. Post-operative external beam radiation after STR is generally accepted management, and estimated five-year PFS ranges from 40 to 90% ^42^. SRS after STR may have a tumor control similar to that of external beam radiation; however, SRS is more beneficial for smaller meningiomas ^39, 49, 50^. A prospective phase II trial (ClinicalTrials.gov identifier: NCT00895622) that was conducted by the Radiation Therapy Oncology Group and that studied the effects of post-operative RT in intermediate-grade meningiomas (recurrent grade I with any extent of resection and gross totally resected grade II meningioma) reported a three-year PFS of 96% in patients who received RT ^23^. A prospective NRG trial (ClinicalTrials.gov identifier: NCT03180268) is under way to study observation compared with radiation in patients with newly diagnosed gross totally resected WHO grade II meningiomas.

Management for MMs (WHO grade III) also consists of surgery with a goal of maximal safe resection. The five-year PFS rates are 28% after GTR and 0% after STR ^51^. Based on a few retrospective analyses, adjuvant radiation demonstrated improved PFS and OS compared with surgery alone ^52, 53^. Post-operative external beam radiation should be performed after any extent of resection for WHO grade III meningiomas ^42^.

There are limited chemotherapy options for meningiomas. According to National Comprehensive Cancer Network guidelines, alpha interferon, somatostatin receptor agonists, and VEGF inhibitor are the only classes of recommended drugs and have previously shown only modest benefit ^54– 56^. Numerous agents such as tyrosine kinase inhibitors, especially epidermal growth factor receptor (EGFR) inhibitors, hydroxyurea, traditional cytotoxic chemotherapy, and hormone receptor–targeted agents, have been studied and have not shown any appreciable impact on survival. However, much of this data is based on retrospective analysis or small phase II trials rather than controlled prospective trials ^57^. Kaley *et al*. have comprehensively reviewed the literature for medical therapies for surgery and radiation-refractory meningioma which revealed significant heterogeneity in study design, criteria for monitoring and progression, and patient selection ^58^. Future trials would greatly benefit from standardization of reporting prior therapies, pre-treatment growth rate, and PFS and OS ^58^.

Bevacizumab, an anti-angiogenic VEGF inhibitor, is a commonly used agent and its use as monotherapy for recurrent meningiomas is being studied (ClinicalTrials.gov identifier: NCT01125046). Results from the CEVOREM (Combination of Everolimus and Octreotide LAR in Aggressive Recurrent Meningiomas) trial (ClinicalTrials.gov identifier: NCT02333565), which studied the combination of octreotide (somastatin analogue) and everolimus (mTOR inhibitor) in recurrent WHO I–III meningiomas, showed activity with acceptable and manageable toxicity. Six-month PFS was almost 60% and in some patients there was a decrease in growth rate of greater than 50 ^59^. Trabectedin, a DNA binding alkylating agent, is being tested in a phase II EORTC (European Organisation for Research and Treatment of Cancer) study (ClinicalTrials.gov identifier: NCT02234050) which is assessing the activity, toxicity, and quality of life in patients with AM and MM. There are two ongoing trials for recurrent AM and MM using Optune, a device worn on the scalp which uses alternating electrical fields, with and without bevacizumab (ClinicalTrials.gov identifiers: NCT01892397 and NCT02847559).

An improved understanding of meningioma biology has led to the study of several novel targets. There have been promising responses in preclinical studies such as with pegvisomant, a growth hormone receptor antagonist; valproic acid as a radiosensitizer and apoptic marker upregulator; a combination of tumor necrosis factor–related apoptosis-inducing ligand (TRAIL, a cytokine that binds to death receptors) and bortezomib (a proteasome inhibitor); and amino levulinic acid (5-ALA) as a photosensitizing agent ^45, 60– 62^. An Alliance consortium group trial (ClinicalTrials.gov identifier: NCT02523014) is currently investigating targeted treatments in progressive meningioma on the basis of mutational status ( *NF2*, *AKT*, and *SMO*).

## Conclusions

There have been numerous advances in our understanding of meningiomas and minor refinements in their diagnosis and management; however, we are still limited in our ability to predict recurrence and there are only a few medical treatment options. There are significant ongoing efforts to further understand the molecular, genetic, epigenetic basis of meningiomas which will undoubtedly revolutionize the classification system with important implications for diagnosis, prognosis, and therapeutics and trial design in the future.

## References

[1] Czyz M Radwan H Li JY: Fractal Analysis May Improve the Preoperative Identification of Atypical Meningiomas. *Neurosurgery.* 2017;80(2):300–8. 10.1093/neuros/nyw030 28173535

[2] Hwang WL Marciscano AE Niemierko A: Imaging and extent of surgical resection predict risk of meningioma recurrence better than WHO histopathological grade. *Neuro Oncol.* 2016;18(6):863–72. 10.1093/neuonc/nov285 26597949 PMC4864259

[3] Ostrom QT Gittleman H Liao P: CBTRUS Statistical Report: Primary brain and other central nervous system tumors diagnosed in the United States in 2010-2014. *Neuro Oncol.* 2017;19(suppl_5):v1–v88. 10.1093/neuonc/nox158 29117289 PMC5693142

[4] Sahm F Schrimpf D Stichel D: DNA methylation-based classification and grading system for meningioma: a multicentre, retrospective analysis. *Lancet Oncol.* 2017;18(5):682–94. 10.1016/S1470-2045(17)30155-9 28314689

[5] Sanson M Kalamarides M: Epigenetics: a new tool for meningioma management? *Lancet Oncol.* 2017;18(5):569–70. 10.1016/S1470-2045(17)30153-5 28314686

[6] Bi WL Abedalthagafi M Horowitz P: Genomic landscape of intracranial meningiomas. *J Neurosurg.* 2016;125(3):525–35. 10.3171/2015.6.JNS15591 26771848

[7] Stamenkovic I Yu Q: Merlin, a "magic" linker between extracellular cues and intracellular signaling pathways that regulate cell motility, proliferation, and survival. *Curr Protein Pept Sci.* 2010;11(6):471–84. 10.2174/138920310791824011 20491622 PMC2946555

[8] Striedinger K VandenBerg SR Baia GS: The neurofibromatosis 2 tumor suppressor gene product, merlin, regulates human meningioma cell growth by signaling through YAP. *Neoplasia.* 2008;10(11):1204–12. 10.1593/neo.08642 18953429 PMC2570596

[9] Xu HM Gutmann DH: Merlin differentially associates with the microtubule and actin cytoskeleton. *J Neurosci Res.* 1998;51(3):403–15. 10.1002/(SICI)1097-4547(19980201)51:3<403::AID-JNR13>3.0.CO;2-7 9486775

[10] Peyrard M Fransson I Xie YG: Characterization of a new member of the human beta-adaptin gene family from chromosome 22q12, a candidate meningioma gene. *Hum Mol Genet.* 1994;3(8):1393–9. 10.1093/hmg/3.8.1393 7987321

[11] Schmitz U Mueller W Weber M: INI1 mutations in meningiomas at a potential hotspot in exon 9. *Br J Cancer.* 2001;84(2):199–201. 10.1054/bjoc.2000.1583 11161377 PMC2363707

[12] Mawrin C Perry A: Pathological classification and molecular genetics of meningiomas. *J Neurooncol.* 2010;99(3):379–91. 10.1007/s11060-010-0342-2 20809251

[13] Olar A Wani KM Wilson CD: Global epigenetic profiling identifies methylation subgroups associated with recurrence-free survival in meningioma. *Acta Neuropathol.* 2017;133(3):431–44. 10.1007/s00401-017-1678-x 28130639 PMC5600514

[14] Ragel BT Jensen RL: Molecular genetics of meningiomas. *Neurosurg Focus.* 2005;19(5):E9. 10.3171/foc.2005.19.5.10 16398473

[15] Mei Y Bi WL Greenwald NF: Genomic profile of human meningioma cell lines. *PLoS One.* 2017;12(5):e0178322. 10.1371/journal.pone.0178322 28552950 PMC5446134

[16] Yaswen P MacKenzie KL Keith WN: Therapeutic targeting of replicative immortality. *Semin Cancer Biol.* 2015;35 Suppl:S104–S128. 10.1016/j.semcancer.2015.03.007 25869441 PMC4600408

[17] Senol O Schaaij-Visser TB Erkan EP: miR-200a-mediated suppression of non-muscle heavy chain IIb inhibits meningioma cell migration and tumor growth *in vivo*. *Oncogene.* 2015;34(14):1790–8. 10.1038/onc.2014.120 24858044

[18] Wang M Deng X Ying Q: MicroRNA-224 targets ERG2 and contributes to malignant progressions of meningioma. *Biochem Biophys Res Commun.* 2015;460(2):354–61. 10.1016/j.bbrc.2015.03.038 25783051

[19] Ludwig N Kim YJ Mueller SC: Posttranscriptional deregulation of signaling pathways in meningioma subtypes by differential expression of miRNAs. *Neuro Oncol.* 2015;17(9):1250–60. 10.1093/neuonc/nov014 25681310 PMC4588755

[20] Tang M Wei H Han L: Whole-genome sequencing identifies new genetic alterations in meningiomas. *Oncotarget.* 2017;8(10):17070–80. 10.18632/oncotarget.15043 28177878 PMC5370023

[21] Kim BW Kim MS Kim SW: Peritumoral brain edema in meningiomas : Correlation of radiologic and pathologic features. *J Korean Neurosurg Soc.* 2011;49(1):26–30. 10.3340/jkns.2011.49.1.26 21494359 PMC3070891

[22] Hou J Kshettry VR Selman WR: Peritumoral brain edema in intracranial meningiomas: the emergence of vascular endothelial growth factor-directed therapy. *Neurosurg Focus.* 2013;35(6):E2. 10.3171/2013.8.FOCUS13301 24289127

[23] Wang C Kaprealian TB Suh JH: Overall survival benefit associated with adjuvant radiotherapy in WHO grade II meningioma. *Neuro Oncol.* 2017;19(9):1263–70. 10.1093/neuonc/nox007 28371851 PMC5570224

[24] Amer ME Heo MS Brooks SL: Anatomical variations of trabecular bone structure in intraoral radiographs using fractal and particles count analyses. *Imaging Sci Dent.* 2012;42(1):5–12. 10.5624/isd.2012.42.1.5 22474642 PMC3314838

[25] Copley SJ Giannarou S Schmid VJ: Effect of aging on lung structure *in vivo*: assessment with densitometric and fractal analysis of high-resolution computed tomography data. *J Thorac Imaging.* 2012;27(6):366–71. 10.1097/RTI.0b013e31825148c9 22487994

[26] Di Ieva A Esteban FJ Grizzi F: Fractals in the neurosciences, Part II: clinical applications and future perspectives. *Neuroscientist.* 2015;21(1):30–43. 10.1177/1073858413513928 24362814

[27] Sciuto R Ferraironi A Semprebene A: Clinical relevance of 111In-octreotide scans in CNS tumors. *Q J Nucl Med.* 1995;39(4 suppl 1):101–3. 9002762

[28] Sommerauer M Burkhardt JK Frontzek K: 68Gallium-DOTATATE PET in meningioma: A reliable predictor of tumor growth rate? *Neuro Oncol.* 2016;18(7):1021–7. 10.1093/neuonc/now001 26865086 PMC4896546

[29] Simpson D: The recurrence of intracranial meningiomas after surgical treatment. *J Neurol Neurosurg Psychiatry.* 1957;20(1):22–39. 10.1136/jnnp.20.1.22 13406590 PMC497230

[30] Gallagher MJ Jenkinson MD Brodbelt AR: WHO grade 1 meningioma recurrence: Are location and Simpson grade still relevant? *Clin Neurol Neurosurg.* 2016;141:117–21. 10.1016/j.clineuro.2016.01.006 26780494

[31] Mathiesen T Gerlich A Kihlström L: Effects of using combined transpetrosal surgical approaches to treat petroclival meningiomas. *Neurosurgery.* 2007;60(6):982–91; discussion 991–2. 10.1227/01.NEU.0000255476.06247.F1 17538371

[32] Pollock BE: Comment on stereotactic radiosurgery for meningioma. In: editor. Contemporary stereotactic radiosurgery: technique and evaluation. Armonk (NY): Futura Publishing.2002;172–80.

[33] Nakamura M Roser F Michel J: The natural history of incidental meningiomas. *Neurosurgery.* 2003;53(1):62–70; discussion 70–1. 10.1227/01.NEU.0000068730.76856.58 12823874

[34] Stafford SL Perry A Suman VJ: Primarily resected meningiomas: outcome and prognostic factors in 581 Mayo Clinic patients, 1978 through 1988. *Mayo Clin Proc.* 1998;73(10):936–42. 10.4065/73.10.936 9787740

[35] Glaholm J Bloom HJ Crow JH: The role of radiotherapy in the management of intracranial meningiomas: the Royal Marsden Hospital experience with 186 patients. *Int J Radiat Oncol Biol Phys.* 1990;18(4):755–61. 10.1016/0360-3016(90)90394-Y 2108937

[36] Lee JY Niranjan A McInerney J: Stereotactic radiosurgery providing long-term tumor control of cavernous sinus meningiomas. *J Neurosurg.* 2002;97(1):65–72. 10.3171/jns.2002.97.1.0065 12134934

[37] Iwai Y Yamanaka K Ikeda H: Gamma Knife radiosurgery for skull base meningioma: long-term results of low-dose treatment. *J Neurosurg.* 2008;109(5):804–10. 10.3171/JNS/2008/109/11/0804 18976068

[38] Pollock BE Stafford SL Utter A: Stereotactic radiosurgery provides equivalent tumor control to Simpson Grade 1 resection for patients with small- to medium-size meningiomas. *Int J Radiat Oncol Biol Phys.* 2003;55(4):1000–5. 10.1016/S0360-3016(02)04356-0 12605979

[39] Cohen-Inbar O Lee CC Sheehan JP: The Contemporary Role of Stereotactic Radiosurgery in the Treatment of Meningiomas. *Neurosurg Clin N Am.* 2016;27(2):215–28. 10.1016/j.nec.2015.11.006 27012386

[40] Kondziolka D Mathieu D Lunsford LD: Radiosurgery as definitive management of intracranial meningiomas. *Neurosurgery.* 2008;62(1):53–8; discussion 58-60. 10.1227/01.NEU.0000311061.72626.0D 18300891

[41] Kreil W Luggin J Fuchs I: Long term experience of gamma knife radiosurgery for benign skull base meningiomas. *J Neurol Neurosurg Psychiatry.* 2005;76(10):1425–30. 10.1136/jnnp.2004.049213 16170090 PMC1739368

[42] Sun SQ Hawasli AH Huang J: An evidence-based treatment algorithm for the management of WHO Grade II and III meningiomas. *Neurosurg Focus.* 2015;38(3):E3. 10.3171/2015.1.FOCUS14757 25727225

[43] Choi Y Lim DH Jo K: Efficacy of postoperative radiotherapy for high grade meningiomas. *J Neurooncol.* 2014;119(2):405–12. 10.1007/s11060-014-1507-1 24965339

[44] Hammouche S Clark S Wong AH: Long-term survival analysis of atypical meningiomas: survival rates, prognostic factors, operative and radiotherapy treatment. *Acta Neurochir (Wien).* 2014;156(8):1475–81. 10.1007/s00701-014-2156-z 24965072

[45] Hanakita S Koga T Igaki H: Role of gamma knife surgery for intracranial atypical (WHO grade II) meningiomas. *J Neurosurg.* 2013;119(6):1410–4. 10.3171/2013.8.JNS13343 24074490

[46] Goyal LK Suh JH Mohan DS: Local control and overall survival in atypical meningioma: a retrospective study. *Int J Radiat Oncol Biol Phys.* 2000;46(1):57–61. 10.1016/S0360-3016(99)00349-1 10656373

[47] Jenkinson MD Waqar M Farah JO: Early adjuvant radiotherapy in the treatment of atypical meningioma. *J Clin Neurosci.* 2016;28:87–92. 10.1016/j.jocn.2015.09.021 26775147

[48] Aghi MK Carter BS Cosgrove GR: Long-term recurrence rates of atypical meningiomas after gross total resection with or without postoperative adjuvant radiation. *Neurosurgery.* 2009;64(1):56–60; discussion 60. 10.1227/01.NEU.0000330399.55586.63 19145156

[49] Hardesty DA Wolf AB Brachman DG: The impact of adjuvant stereotactic radiosurgery on atypical meningioma recurrence following aggressive microsurgical resection. *J Neurosurg.* 2013;119(2):475–81. 10.3171/2012.12.JNS12414 23394332

[50] Sun SQ Cai C Murphy RK: Management of atypical cranial meningiomas, part 2: predictors of progression and the role of adjuvant radiation after subtotal resection. *Neurosurgery.* 2014;75(4):356–63; discussion 363. 10.1227/NEU.0000000000000462 24932708

[51] Dziuk TW Woo S Butler EB: Malignant meningioma: an indication for initial aggressive surgery and adjuvant radiotherapy. *J Neurooncol.* 1998;37(2):177–88. 10.1023/A:1005853720926 9524097

[52] Zhao P Hu M Zhao M: Prognostic factors for patients with atypical or malignant meningiomas treated at a single center. *Neurosurg Rev.* 2015;38(1):101–7; discussion 107. 10.1007/s10143-014-0558-2 25139398

[53] Durand A Labrousse F Jouvet A: WHO grade II and III meningiomas: a study of prognostic factors. *J Neurooncol.* 2009;95(3):367–75. 10.1007/s11060-009-9934-0 19562258

[54] Chamberlain MC Glantz MJ Fadul CE: Recurrent meningioma: salvage therapy with long-acting somatostatin analogue. *Neurology.* 2007;69(10):969–73. 10.1212/01.wnl.0000271382.62776.b7 17785665

[55] Kaley TJ Wen P Schiff D: Phase II trial of sunitinib for recurrent and progressive atypical and anaplastic meningioma. *Neuro Oncol.* 2015;17(1):116–21. 10.1093/neuonc/nou148 25100872 PMC4483051

[56] Chamberlain MC Glantz MJ: Interferon-alpha for recurrent World Health Organization grade 1 intracranial meningiomas. *Cancer.* 2008;113(8):2146–51. 10.1002/cncr.23803 18756531

[57] Karsy M Guan J Cohen A: Medical Management of Meningiomas: Current Status, Failed Treatments, and Promising Horizons. *Neurosurg Clin N Am.* 2016;27(2):249–60. 10.1016/j.nec.2015.11.002 27012389

[58] Kaley T Barani I Chamberlain M: Historical benchmarks for medical therapy trials in surgery- and radiation-refractory meningioma: a RANO review. *Neuro Oncol.* 2014;16(6):829–40. 10.1093/neuonc/not330 24500419 PMC4022224

[59] Paldor I Awad M Sufaro YZ: Review of controversies in management of non-benign meningioma. *J Clin Neurosci.* 2016;31:37–46. 10.1016/j.jocn.2016.03.014 27338209

[60] McCutcheon IE Flyvbjerg A Hill H: Antitumor activity of the growth hormone receptor antagonist pegvisomant against human meningiomas in nude mice. *J Neurosurg.* 2001;94(3):487–92. 10.3171/jns.2001.94.3.0487 11235955

[61] Koschny R Boehm C Sprick MR: Bortezomib sensitizes primary meningioma cells to TRAIL-induced apoptosis by enhancing formation of the death-inducing signaling complex. *J Neuropathol Exp Neurol.* 2014;73(11):1034–46. 10.1097/NEN.0000000000000129 25289891

[62] El-Khatib M Tepe C Senger B: Aminolevulinic acid-mediated photodynamic therapy of human meningioma: an *in vitro* study on primary cell lines. *Int J Mol Sci.* 2015;16(5):9936–48. 10.3390/ijms16059936 25941934 PMC4463626

